# The effects of chronotherapy for hypertension on cardiovascular outcomes: A commentary of current knowledge and future directions

**DOI:** 10.1016/j.amsu.2020.09.037

**Published:** 2020-10-01

**Authors:** Rahul Ramesh Rana, Sidharth Sunil menon

**Affiliations:** University College London Medical School, 74 Huntley St, Bloomsbury, London, WC1E 6DE, United Kingdom

**Keywords:** Chronotherapy, Hypertension, Antihypertensives, Cardiovascular, Hygia

Dear editor,

A concept as simple as chronotherapy possessing the potential to improve the cardiovascular outcomes of hypertensive patients, with zero cost, acutely fascinated us. The recent 2019 Hygia trial incited this interest and prompted us to write this letter, in which we aim to highlight and discuss the results of this trial and its implications on the field of chronotherapy.

Systemic arterial hypertension (HTN), arguably the most prominent risk factor for cardiovascular disease (CVD), is characterised by a consistent systolic and diastolic blood pressure of 140 and 90 or above respectively [1]. Blood pressure (BP) fluctuates throughout the day, however, is relatively stable and generally lower at night. This night-time drop in BP, also referred to as the nocturnal BP dip, is related to the circadian rhythm of the cardiovascular system [2]. On average, BP drops by 10–20% while asleep. An individual with nocturnal BP dip of less than 10% is considered to be ‘non-dipping’ [2]. Individuals with HTN are more likely to have a non-dipping presentation [3]. Non-dipping individuals are at a greater cardiovascular risk than normal dipping individuals [3]. This finding is supported with additional research, which state that night-time ambulatory blood pressure monitoring (ABPM) is a better prognostic determinant for cardiovascular and all-cause mortality compared to day-time ABPM [4,5].

The rationale behind chronotherapy is to aim to reduce night-time BP by administering anti-hypertensives at night, diminishing the non-dipping abnormality.

Results from the 1997 Syst-Eur trial and the 2000 HOPE trial indicated the ability of chronotherapy to decrease the frequency of the ‘non-dipping’ pattern by decreasing mean night-time BP [6,7]. However, the first long term clinical trial which was specifically created to test the effects of chronotherapy on CVD outcomes in hypertensive patients was the MAPEC study. Published in 2010, the results of the study were concordant to previous studies, discovering a decrease in night-time BP when administering anti-hypertensives at night and better CVD outcomes after a 5.7 year follow up [8]. After the promising results obtained from the MAPEC trial, an improved trial with a larger sample size was warranted. This came in the form of the Hygia trial.

Published in late 2019, the Hygia Chronotherapy trial is the largest and latest study of the topic to date [9]. The population size consisted of 19,084 patients with a mean age of 60.5. The intervention group consumed ≥1 anti-hypertensive/s prior to sleep, whilst the control group ingested them upon awakening. Anti-hypertensives of the following classes were prescribed: ARB, ACEi, CCB, beta-blockers and/or diuretics.

Ensuing an average follow-up time of 6.3 years, a significant decrease in the prevalence of primary CVD outcome (myocardial infarction, coronary revascularisation, stroke, CVD death and heart failure) was seen in the intervention group in comparison to the control group (0.55 hazard ratio (HR) [95% confidence interval 0.50 to 0.61]; P < 0.001), with similarly reduced HR in all different types of CVD (p < 0.001 in every case). The intervention also considerably reduced the frequency of non-dipping patterns in patients alongside decreasing mean night-time ambulatory BP. This data supports previous literature, which correlates a lower prevalence of non-dipping to better CVD outcomes. Baseline characteristics were similar between the 2 groups hence randomisation was successful. No negative effects of the intervention were observed. Quality control was maintained through constant observation by 292 trained investigators who conducted regular audits to monitor quality and adherence to the inclusion/exclusion criteria and ethical standard, suggesting the data is of high quality. The sample size in the Hygia trial was larger than MAPEC by a factor of 8.85 and has a longer duration of follow up, Hygia is more statistically powerful.

It is worth mentioning chronic kidney disease (CKD), which is a major cause of HTN. HTN is present in 90% of patients with severe CKD and hypertensive individuals with impaired renal function tend to have a higher systolic BP compared to individuals with normal renal function [10]. More importantly, CKD is associated with a reduced dipping BP pattern at night, explaining the high CVD risk profiles of these individuals. Chronotherapy is extremely effective in patients with CKD. Data obtained by R C Hermida et al. displayed a substantial reduction of 69% in CVD events after a follow up of 5.4 years (0.31 HR [95% confidence interval 0.21 to 0.46]; P < 0.001) in patients with CKD and HTN who ingested their anti-hypertensives at night, as well as a reduction in non-dipping patterns. This enhances the credibility of this treatment option, in particular for patients with CKD, considering its morbid cyclic relationship with HTN [11].

Despite the promising evidence in favour of the intervention, present clinical guidelines still advise taking anti-hypertensives upon awakening. There is a consensus that more evidence is yet required if the current clinical guidelines are to be changed. The biggest limitation of the Hygia and MAPEC trials must be addressed in future trials, a more diverse study population is vital to test the effects of the intervention on individuals of all ethnicities. The cost-free and as research has shown, ‘safe’ nature of this intervention makes a much larger global trial feasible. Bedtime ingestion of anti-hypertensives as a concept is gaining creditability due to trials such as Hygia; with sufficient further evidence, the clinical guidelines are likely to change in favour of this intervention.

## Ethical approval

No ethical approval was required.

## Sources of funding

No funding. All resources were available online.

## Author contribution

Mr. Rahul Ramesh Rana was the lead author of this particular letter. Mr. Sidharth Sunil Menon was also an author of this letter, performing data analysis of the mentioned studies.

## Registration of research studies

1 Name of the registry: N/A.
2 Unique Identifying number or registration ID: N/A.
3 Hyperlink to your specific registration (must be publicly accessible and will be checked): N/A.

## Guarantor

Mr. Rahul Ramesh Rana and Mr. Sidharth Sunil Menon approve to take full responsibility of this commentary.

## Provenance and peer review

Not commissioned, externally peer reviewed.

## Declaration of competing interest

None.
